# Minimally invasive or open esophagectomy for esophageal squamous cell carcinoma: a comprehensive systematic review of surgical and survival outcomes

**DOI:** 10.3389/fsurg.2026.1734948

**Published:** 2026-02-17

**Authors:** Anas B. Barnawi, Waseem M. Hajjar, Adel D. Almaymuni, Ammar Alzahim, Osama Thamer Al-Ahmari, Basim Alshahrani, Abdulaziz Aljanoubi, Layan Rafat Bukhari, Muhanad Sultan Alsharari, Meshari Abdulrahman Al-Sahli, Abdulmalik Abdulelah Bin Kassim, Aldana Alodayani

**Affiliations:** 1 College of Medicine, Imam Mohammad Ibn Saud Islamic University (IMSIU), Riyadh, Saudi Arabia; 2 Department of Surgery, College of Medicine, King Saud University, Riyadh, Saudi Arabia; 3 Department of Surgery, King Fahad Armed Forces Hospital, Jeddah, Saudi Arabia; 4 King Saud University Medical City, Riyadh, Saudi Arabia

**Keywords:** esophageal squamous cell carcinoma, minimally invasive esophagectomy, oncologic outcomes, open esophagectomy, overall survival, postoperative recovery, pulmonary complications, systematic review

## Abstract

**Background:**

Esophageal squamous cell carcinoma (ESCC) remains a common malignancy with high mortality. Minimally invasive esophagectomy (MIE) was developed to reduce the morbidity of conventional open esophagectomy (OE), but comparative evidence specifically addressing oncologic adequacy and postoperative recovery in ESCC is limited. This systematic review synthesizes comparative data on MIE vs. OE in ESCC.

**Methods:**

We conducted a PRISMA-compliant systematic review registered on PROSPERO (CRD420251158559). PubMed/MEDLINE, Web of Science, and the Cochrane Library were searched for studies published between January 2010 and May 2024. Nine comparative studies (*n* = 5,342; 2,968 MIE, 2,374 OE) met inclusion criteria. Methodological quality was assessed using the Newcastle–Ottawa Scale. Prespecified endpoints included overall survival (OS), disease-free survival (DFS), lymph node yield, R0 resection rate, perioperative complications, intraoperative blood loss, and lengths of ICU and hospital stay.

**Results:**

Aggregate data indicate oncologic equivalence between MIE and OE: R0 resection rates were uniformly high (≥92%), and lymph node yields were comparable. Five out of nine studies (55.6%) reported no statistically significant differences in overall survival (OS) or disease-free survival (DFS) between MIE and OE. However, selected analyses favored MIE (e.g., 3-year OS HR 0.54, 95% CI 0.43–0.68). Perioperatively, MIE demonstrated consistent advantages, including reduced intraoperative blood loss, shorter hospital length of stay, and lower rates of pulmonary complications—particularly pneumonia—each of which was reported in seven of the nine included studies (77.8%). Anastomotic leak rates were similar; reports of recurrent laryngeal nerve injury were heterogeneous.

**Conclusion:**

In ESCC, MIE achieves oncologic outcomes comparable to OE while conferring reduced pulmonary morbidity, lower blood loss, and accelerated postoperative recovery, supporting its consideration as a standard surgical approach.

**Systematic Review Registration:**

https://www.crd.york.ac.uk/PROSPERO/view/CRD420251158559, PROSPERO CRD420251158559.

## Introduction

Esophageal squamous cell carcinoma (ESCC) remains a major global health challenge. In 2020, there were ∼604,000 new esophageal cancers and ∼544,000 deaths worldwide, and roughly 85% of these tumors were squamous cell carcinomas (1). Incidence and mortality are highly concentrated in East Asia and parts of Africa (1). Notably, esophageal cancer is extremely lethal—contemporary estimates place 5-year survival well below 25%, reflecting the fact that many cases present at advanced stages (1, 2). Although ESCC predominates in high-risk regions (where as much as half of global ESCC occurs, e.g., China), its patterns vary by geography and risk factors (3). In Western countries, the proportion of ESCC has declined in favor of adenocarcinoma, but worldwide, squamous histology still accounts for most cases (1, 2). Because ESCC often presents as locally advanced disease, multimodal therapy (typically neoadjuvant chemoradiotherapy followed by surgery) is standard practice (2, 3). Radical esophagectomy with lymphadenectomy is considered the mainstay of curative treatment for resectable ESCC (3, 4).

Traditional open esophagectomy (OE)—most commonly an Ivor Lewis or McKeown procedure via right thoracotomy and laparotomy—has long been the standard operative approach. However, open esophagectomy is technically demanding and associated with substantial morbidity and mortality. Even in modern series, perioperative mortality remains on the order of 2%–5%, and overall complication rates approach 50%–60% (4, 5). Pulmonary complications are common: historical data suggest respiratory events in roughly half of patients undergoing OE (5). Patients also endure significant blood loss, extensive pain, and prolonged intensive care and hospitalization following an open thoracotomy. These limitations have motivated efforts to reduce the “surgical stress” of esophagectomy while maintaining oncologic effectiveness (4, 5). For example, high-volume centers and standardized perioperative pathways have improved outcomes somewhat; however, the intrinsic invasiveness of open resection, including large incisions and one-lung ventilation, still carries a high risk. Definitive chemoradiation can be curative for some SCC patients, but for most respectable tumors, operative resection remains necessary (3, 5).

Starting in the 1990s, minimally invasive esophagectomy (MIE) techniques were introduced to address these challenges. The pioneering work of Cuschieri and others showed that esophagectomy could be done via thoracoscopic and laparoscopic access (5). Over the past decade, these approaches have been adopted widely, with variations including total MIE (combined thoracoscopic and laparoscopic Ivor Lewis or McKeown), hybrid techniques (e.g., laparoscopic abdomen with open chest or vice versa), and robotic-assisted procedures (4, 5). In practice, an Ivor Lewis esophagectomy can now be performed in three major ways: (1) OE via thoracotomy plus laparotomy; (2) total MIE via thoracoscopy and laparoscopy; or (3) hybrid (HE) combining an open thoracic phase with a laparoscopic abdominal phase (or the reverse) (4). Robotic-assisted MIE (RAMIE) is a more recent innovation, offering enhanced dexterity for esophageal dissection. These minimally invasive strategies aim to spare healthy tissue and reduce operative trauma, thereby enhancing postoperative recovery (4, 5). By minimizing chest wall and diaphragmatic incisions, MIE/HE approaches seek to lower pulmonary complications and accelerate the return of function without compromising oncologic principles (4, 5).

Comparative studies indicate that MIE generally confers perioperative advantages over OE. Multiple meta-analyses and trials have reported that MIE (including hybrid and minimally invasive techniques) significantly reduces respiratory complications, blood loss, and intensive care and hospital length of stay (5, 6). For example, a 2020 meta-analysis of over 13,000 patients found that MIE was associated with a markedly lower odds of any respiratory complication (odds ratio ≈0.56), as well as shorter hospital stays and less intraoperative bleeding. However, operative time was longer with MIE (6). Similarly, the TIME randomized trial (laparoscopy + thoracoscopy vs. open) demonstrated significantly fewer pulmonary infections and shorter hospitalization with MIE, along with better short-term quality-of-life scores (4, 5). Retrospective registry analyses also support these trends: a large U.S. surgical database review showed consistently lower rates of pulmonary and other complications and shorter lengths of stay for MIE compared to open, across various operative techniques (4). In sum, current evidence suggests that patients undergoing minimally invasive esophagectomy experience less postoperative pain, earlier mobilization, and quicker functional recovery than those having traditional open surgery (5).

Importantly, these perioperative benefits have so far not come at the expense of cancer control. Resection margins and lymph node yields appear comparable between MIE and OE. Multiple series have shown that MIE achieves R0 resection rates and nodal harvests that are at least as good as open surgery (4, 5). Concerning long-term outcomes, many analyses report equivalent overall survival with MIE, and some suggest it may even improve survival. A large multicenter analysis (ENSURE trial secondary analysis) found that minimally invasive Ivor Lewis (thoracoscopic/laparoscopic) was independently associated with better disease-free survival, and that both hybrid and total MIE had superior overall survival compared with open esophagectomy (7). Prior systematic reviews likewise observed that overall and cancer-specific survival after MIE was at least non-inferior and possibly superior to open surgery (5, 6). Patients who had MIE show similar long-term recurrence rates; the enhanced visualization and meticulous lymphadenectomy possible with minimally invasive techniques may even improve oncologic quality of surgery (5, 7). Thus far, no convincing evidence has emerged that the minimally invasive approach compromises oncologic outcomes, and accumulating data hint that the less invasive approach could marginally benefit survival, perhaps by allowing more patients to complete neoadjuvant therapy and adjuvant regimens.

Quality-of-life (QoL) after esophagectomy is a critical patient-centered outcome. Studies consistently report that MIE patients experience better short-term QoL, with less pain, fatigue, and physical dysfunction, particularly in the first few months after surgery (5, 8). For example, one meta-analysis found that MIE patients had significantly higher global QoL and better physical function at 3 months postoperatively (but by 6–12 months, most differences had dissipated) (8). Randomized data also show improved early postoperative QoL with MIE: in the TIME trial, the physical components of health-related QoL scores at 6 weeks favored the minimally invasive group (4, 5). By one year after surgery, most studies find little difference in global QoL between approaches, although some reports indicate persistent advantages in pain or symptom domains for MIE (8). In practical terms, the reduced pain and quicker recovery seen with MIE can translate into a faster return to normal activities and work. Economic analyses are evolving: some cost-effectiveness studies suggest that MIE's higher operative costs (especially for robotic systems) may be largely offset by shorter hospital stays and fewer complications (4). Notably, the recent ROMIO trial found no significant overall cost difference between hybrid (laparoscopic plus thoracotomy) and open esophagectomy, implying that the two approaches may be roughly equivalent economically when amortized over all postoperative care (4). In practice, any “extra” cost of minimally invasive equipment must be weighed against system savings from reduced ICU time and complications (4).

Given the rapid technical evolution and the growing body of evidence, a rigorous updated synthesis is urgently needed. While previous systematic reviews and meta-analyses (e.g., Akhtar et al., 2020) have established short-term benefits of MIE over OE, most of these studies included mixed histological types—primarily combining squamous cell carcinoma with adenocarcinoma—despite their differing biological behavior and treatment responses (6). Esophageal squamous cell carcinoma (ESCC), which remains the predominant histology globally, especially in high-incidence regions like East Asia, has unique epidemiologic and therapeutic considerations. Importantly, no comprehensive, up-to-date systematic review has focused exclusively on ESCC. Moreover, the endpoints of survival and recovery—specifically overall survival, postoperative complications, pulmonary outcomes, and hospitalization duration—are critical yet variably reported in prior reviews. With accumulating high-quality, histology-specific data, the evolution of advanced MIE techniques (including robotic-assisted approaches), and an international movement toward less invasive oncologic surgery, a focused systematic comparison of MIE vs. OE in esophageal squamous cell carcinoma, emphasizing survival and recovery outcomes, is both timely and clinically essential.

## Methodology

### Literature Search and Study Selection

This systematic review was conducted according to a predefined protocol registered with PROSPERO (CRD420251158559) and adhered to the Preferred Reporting Items for Systematic Reviews (PRISMA) guidelines (9, 10). A comprehensive literature search was performed across PubMed/MEDLINE, Web of Science, and Cochrane Library databases from January 2010 to May 2024. The search strategy employed Medical Subject Headings (MeSH) terms including “Esophageal Squamous Cell Carcinoma,” “Minimally Invasive Surgical Procedures,” and “Esophagectomy,” combined with free-text terms such as (“minimally invasive esophagectomy” OR MIE) AND (“open esophagectomy”) AND (“squamous cell carcinoma” OR ESCC). All identified records were uploaded to Rayyan, a web-based systematic review platform, for collaborative screening (11). Inclusion criteria required comparative studies of MIE vs. OE in ESCC patients with ≥30 patients per group, quantitative outcome reporting (survival, complications, lymph node yield), and English-language publication. Studies were excluded if they involved non-comparative designs, pediatric populations, non-ESCC histology, or techniques predating 2010.

### Screening and Data Extraction

**Figure 1:**
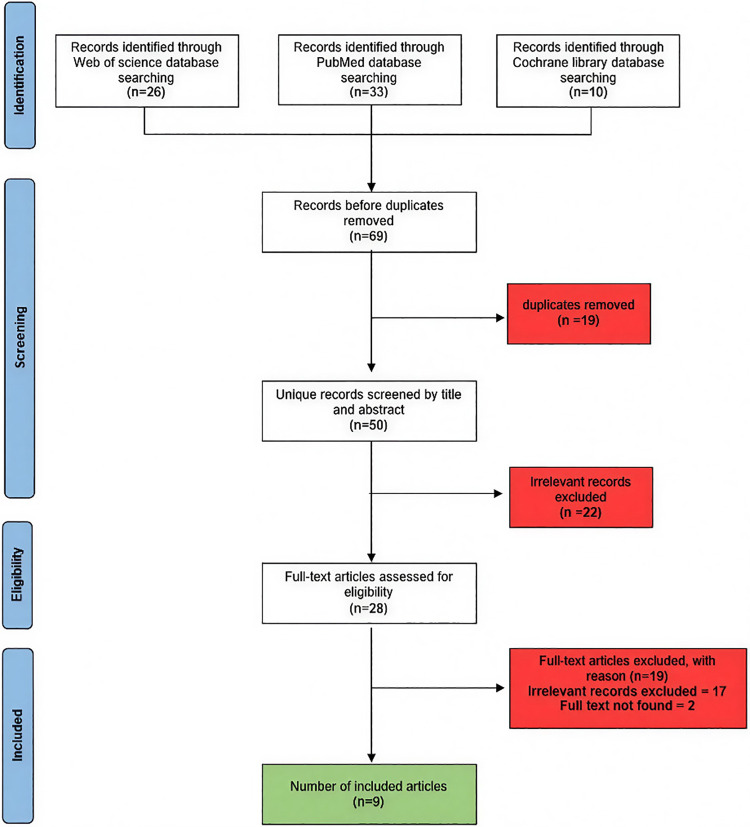
PRISMA flow diagram summarizing the screening process.

The screening process occurred in two phases to ensure methodological rigor. First, two independent reviewers evaluated 69 unique records (after removal of 19 duplicates) by title and abstract. This phase excluded 22 irrelevant studies, leaving 28 articles for full-text assessment. Second, four reviewers independently evaluated these full texts against the inclusion criteria. Seventeen studies were excluded for irrelevance (e.g., mixed histology without subgroup analysis), and two were excluded due to inaccessible full texts despite interlibrary loan requests and author outreach. Nine studies ultimately met the inclusion criteria (12–20). Figure 1 shows the PRISMA flow diagram for the screening process result.

A standardized data extraction form was developed in Microsoft Excel to document study characteristics (author, year, country, design), patient demographics (age, sex, tumor stage), surgical details (MIE/OE approach, conversion rates), and outcomes (survival metrics, complication rates, hospitalization duration). Continuous data were recorded as means ± standard deviation or median [IQR], while categorical outcomes were captured as event counts and percentages. Two reviewers cross-verified all extracted data, with discrepancies resolved through consensus discussions.

### Quality Assessment and Risk of Bias

Methodological quality of the included cohort studies was evaluated using the Newcastle-Ottawa Scale (NOS), which assesses three domains: Selection (case definition, representativeness, comparability group selection), Comparability (adjustment for confounders), and Outcome (ascertainment, follow-up adequacy) (21). Two reviewers independently applied the NOS criteria, awarding stars for each fulfilled item. Studies achieving ≥8 stars were classified as low risk of bias, 6–7 stars as moderate risk, and ≤5 stars as high risk. The evaluation revealed that six studies (Yun 2020, Mao 2023, Yamashita 2018, Wang 2023, Wang 2022, Wang 2015) had low risk of bias (8–9 stars), while three studies (Kanekiyo 2018, Terayama 2024, Hamai 2021) demonstrated moderate risk (6–7 stars) (12–20). Common limitations included unbalanced neoadjuvant therapy between groups (two studies) and insufficient reporting of follow-up completeness (six studies) (Table 1).

**Table 1:** Risk of bias assessment of the included cohort studies using NOS.

Study	Selection				Comparability	Outcome		
	Case Def. adequate?	Representativeness?	Selection of comparison group?	Definition of comparison group?	Comparability of groups?	Outcome ascertainment?	Follow-up: long enough?	Adequacy of follow-up?
Yun et al. (12)	Yes	Yes	Yes	Yes	Yes	Yes	Yes	Yes
Kanekiyo et al. (13)	Yes	Yes	Yes	Yes	Yes	Yes	Yes	(Not reported)
Terayama et al. (15)	Yes	Yes	Yes	Yes	Yes	Yes	Yes	(Not reported)
Mao et al. (15)	Yes	Yes	Yes	Yes	Yes	Yes	Yes	Yes
Yamashita et al. (16)	Yes	Yes	Yes	Yes	Yes	Yes	Yes	(Not reported)
Wang et al. (17)	Yes	Yes	Yes	Yes	Yes	Yes	Yes	(Not reported)
Wang et al. (18)	Yes	Yes	Yes	Yes	Yes	Yes	Yes	(Not reported)
Wang et al. (19)	Yes	Yes	Yes	Yes	Yes	Yes	Yes	(Not reported)
Hamai et al. (20)	Yes	Yes	Yes	Yes	Yes	Yes	Yes	(Not reported)

Study	Selection score	Comparability score	Outcome score	Total score	Risk of bias summary
Yun et al. (12)	⭐⭐⭐⭐	⭐⭐	⭐⭐⭐	9	Low
Kanekiyo et al. (13)	⭐⭐⭐⭐	⭐⭐	⭐	7	Moderate
Terayama et al. (15)	⭐⭐⭐⭐	⭐⭐	⭐	7	Moderate
Mao et al. (15)	⭐⭐⭐⭐	⭐⭐	⭐⭐⭐	9	Low
Yamashita et al. (16)	⭐⭐⭐⭐	⭐⭐	⭐⭐	8	Low
Wang et al. (17)	⭐⭐⭐⭐	⭐⭐	⭐⭐	8	Low
Wang et al. (18)	⭐⭐⭐⭐	⭐⭐	⭐⭐	8	Low
Wang et al. (19)	⭐⭐⭐⭐	⭐⭐	⭐⭐	8	Low
Hamai et al. (20)	⭐⭐⭐⭐	⭐	⭐⭐	7	Moderate

### Characteristics of Included Studies

The nine comparative studies included in this analysis were conducted between 2015 and 2024 across East Asia (China, Japan, Korea, and Taiwan) and comprised 5,342 patients who underwent esophagectomy for esophageal cancer. Of these, 2,968 underwent minimally invasive esophagectomy (MIE) and 2,374 underwent open esophagectomy (OE). Study-level sample sizes ranged from 130 to 1,299 patients. Several investigations applied statistical adjustment methods to mitigate baseline confounding—principally propensity score matching (PSM) and inverse probability of treatment weighting (IPTW)—although the reporting of covariate balance and specific implementation details was heterogeneous. Demographic reporting was inconsistent across reports; where available, the mean/median age clustered at approximately 65 years, and the proportion of male subjects ranged roughly 41%–48%. Reported rates of neoadjuvant therapy (when provided) were high in reporting studies (approximately 80%–98%). Surgical interventions encompassed robotic-assisted, thoracoscopic/laparoscopic (total MIE), hybrid techniques, and conventional open thoracotomy/laparotomy; conversions from minimally invasive to open approaches were infrequently reported and low. Primary oncologic and perioperative endpoints investigated across studies included overall survival (OS), disease-free survival (DFS), lymph-node yield, R0 resection rate, pulmonary and other postoperative complications, and lengths of ICU and hospital stay. Heterogeneous outcome definitions and variable follow-up durations limited direct comparability between reports (Table 2).

**Table 2:** Characteristics of the included studies.

Study (authors, year, journal, country)	Design (Type; funding/COI)	Population (N MIE/OE; age; sex; stage; location; Neoadj; LN field)	Intervention (approach; conversion; operative time; blood loss)	Survival outcomes (OS, DFS; HRs)	Recovery/complications (LN yield, R0%; pulm, leak, RLN; ICU/Hosp stay; QoL)	Methodology (bias, confounders, follow-up, analysis)	Other (30/90d mortality; readmissions)
Yun et al. 2020 (12), Dis Esophagus (Korea)	Type: Retrospective, single-center, observational cohort; Funding: NR (no disclosures); Conflict of Interest: The authors declare no conflicts.	N (MIE/OE): 130/241; Age (mean ± SD): 63.7 ± 8.7 vs. 62.5 ± 8.0; Sex (male%): 90.8 vs. 92.5; Stage (I/II/III%): 73.1/20.0/6.9 vs. 51.0/24.9/24.1; Location (Upper/Mid/Lower%): 34.6/48.5/16.9 vs. 28.6/48.5/22.8; Neoadj: 16.2% vs. 42.3%; LN field: Abdominal (left gastric, celiac, gastrohepatic, pericardial, splenic, peripancreatic, diaphragmatic) + Thoracic (recurrent laryngeal, subcarinal, hilar, azygos, paraesophageal); Extended (bilateral paratracheal).	Approach: RAMIE (da Vinci) thoracic + laparoscopic/robotic gastric conduit (Ivor Lewis or McKeown); OE: Open thoracotomy + laparotomy (Ivor Lewis or McKeown). Conversion: 2.3% (RAMIE→open; OE N/A). Operative time (mean ± SD): 275.6 ± 71.1 vs. 240.0 ± 48.9 min; Blood loss (mean ± SD): 110.8 ± 125.8 vs. 93.8 ± 140.9 mL.	OS: 1-year 95.1% vs. 85.6%; 3-year 81.7% vs. 73.7% (IPTW *P* = 0.051); HR (all-cause mortality): Unadjusted 0.56 (95% CI 0.31–1.01; *P* = 0.054); IPTW-adjusted 0.84 (95% CI 0.47–1.52; *P* = 0.576). DFS: 1-year 54.4% vs. 53.2%; 3-year 49.2% vs. 45.6% (IPTW *P* = 0.217); HR: 0.75 (95% CI 0.35–1.75; *P* = 0.509).	LN yield (mean ± SD): 39.1 ± 13.8 vs. 38.3 ± 12.9; R0 resection: 97.7% vs. 96.7%; Pneumonia: 3.8% vs. 10.8%; Anastomotic leak: 3.1% vs. 2.9%; RLN injury: 25.4% vs. 19.9%; ICU stay (days): 1.08 ± 0.43 vs. 1.36 ± 1.97; Hospital stay (days): 16.5 ± 9.8 vs. 18.2 ± 15.4; QoL: NR.	Bias: Single-center, single-surgeon, retrospective observational (selection bias). Confounders: IPTW (32 variables; SMDs to balance). Follow-up: 1 & 3 months then q6mo up to 5 years (including imaging, death registry). Analysis: *χ*²/Fisher's, *t*-tests; Kaplan–Meier (log-rank), Cox (IPTW-adjusted).	30-day mortality: 0% vs. 1.7%; 90-day: NR; Readmissions: NR.
Kanekiyo et al. 2018 (13), Surg Endosc (Japan)	Type: Retrospective, single-center, propensity-score–matched cohort; Funding: JSPS KAKENHI 24791379; Conflict of Interest: Hazama (NEC, Toyo Kohan); others none.	N (MIE/OE): 65/65 (matched); Age: 66 vs. 66 (median, IQR 62–70 vs. 61–70); Sex (male%): 86.2 vs. 89.2; Stage (path 0–I/II–IV%): 36.9/63.1 vs. 36.9/63.1; Location: NR; Neoadj: 56.9% vs. 53.8%; LN field: 2-field (mediastinal + perigastric) or 3-field (plus cervical).	Approach: TE (thoracoscopic esophagectomy, prone) + HALS laparoscopy; OE: Open right thoracotomy + laparotomy. Conversion: NR. Op time (median, IQR): 536 (501–593) vs. 491 (415–575) min; Blood loss (median, IQR): 250 (160–503) vs. 599 (360–875) mL.	5-year OS: 64.9% vs. 50.2% (*P* = 0.101); 5-year PFS: 70.6% vs. 58.7% (*P* = 0.328). HRs: NR.	LNs (median): 25 vs. 21; R0 resection: NR; Pneumonia: 16.9% vs. 33.9%; Leak: 10.8% vs. 12.3%; RLN palsy: 23.1% vs. 29.3%; ICU stay: NR; Hospital stay (median days): 29 vs. 35; QoL: NR; Other: Significantly lower postoperative IL-6/IL-10 in MIE (less stress).	Bias: Retrospective, non-randomized (potential confounding). Confounders: 1:1 PSM (age, gender, ASA-PS, pathologic stage); Follow-up: exams + CT every 3mo; ∼30.8% reached 5-year. Analysis: Mann–Whitney U, χ²/Fisher; Kaplan–Meier (log-rank).	30-day mortality: 0% vs. 0%; 90-day: NR; Readmissions: NR.
Terayama et al. 2024 (14), Ann Surg Oncol (Japan)	Type: Retrospective, single-center cohort; Funding: NR; Conflict of Interest: none.	N (MIE/OE): 651/382 (IPTW-adjusted); Age (median): 66 vs. 64; Sex (male%): 80.7 vs. 80.8; Tumor stage (cT1/2/3/4%): 47.8/14.8/34.6/2.9 vs. 33.1/25.9/36.5/4.4; Location (U/M/L%): 20.9/44.6/34.5 vs. 18.9/48.4/32.6; Neoadj (%): Chemo 51.1 vs. 52.1; CRT 6.3 vs. 10.3; LN field: NR.	Approach: MIE: Right thoracoscopic + laparoscopic (McKeown or Ivor-Lewis); OE: Open thoracotomy + laparotomy (McKeown or Ivor-Lewis). Conversion: 0.4% (MIE→open); Op time (median, range): 540 (259–957) vs. 492 (260–1,047) min; Blood loss (median, range): 110 (15–880) vs. 380 (50–3,250) mL.	OS: HR (MIE vs. OE) 0.54 (95% CI 0.43–0.68; *P* < 0.001); CSS HR 0.51 (95% CI 0.39–0.67; *P* < 0.001); DFS: NR; cT3–4 subgroup CSS HR 0.65 (95% CI 0.47–0.92; *P* = 0.014).	R0 resection: 98.6% vs. 92.7%; Pneumonia (CD ≥ 2): 18.1% vs. 18.3%; Leak (CD ≥ 2): 10.7% vs. 8.3%; RLN palsy (CD ≥ 2): 4.0% vs. 3.1%; ICU stay: NR; Hospital stay (median days): 20 vs. 22; QoL: NR; Any postop complication (CD ≥ 2): 53.7% vs. 60.8%; Surgical-site infection (CD ≥ 2): 6.6% vs. 11.6%.	Bias: Single-center, retrospective (2005–2021; potential selection). Confounders: IPTW (age, sex, BMI, ASA-PS, preop therapy, tumor location, cT, cN). Follow-up: q3–4mo (yr1), then q6mo; Analysis: Mann–Whitney U, Fisher's exact; Kaplan–Meier, Cox (EZR).	30-day mortality: 0.1% vs. 0.3%; 90-day: NR; Readmissions: NR; Locoregional recurrence HR: 0.48 (95% CI 0.35–0.67; *P* < 0.001); Distant metastasis HR: 0.89 (95% CI 0.60–1.33; *P* = 0.591).
Mao et al. 2023 (15), J Natl Cancer Ctr (China)	Type: Multicenter, prospective, non-randomized cohort; Funding: National Sci & Tech Support (NKTRDP-2015BAI12008-01); Conflict of Interest: none.	N (MIE/OE): 335/335 (PSM); Age (mean ± SD): 61.1 ± 7.2 vs. 60.7 ± 7.2; Sex (male%): 79.4 vs. 78.5; Stage I/II/III%: 12.5/80.3/7.2 vs. 13.4/78.5/8.1; Location (U/M/L%): 33.7/50.4/15.8 vs. 34.3/49.9/15.8; Neoadj: none (no neoadj); LN field: Thoracic (incl. RLNs, subcarinal) + Abdominal.	Approach: MIE: Thoracoscopic (semi-prone) McKeown + laparoscopy; OE: Open thoracotomy + laparotomy + cervical incision (McKeown). Conversion: 0.3% (MIE→open); Op time (mean ± SD): 279 ± 93.5 vs. 277 ± 81.2 min; Blood loss (mean ± SD): 162 ± 196 vs. 227 ± 144 mL.	3-year OS: 77.0% vs. 69.0% (HR 1.33, 95% CI 1.02–1.73; *P* = 0.03); 3-year DFS: 68.1% vs. 60.9% (HR 1.22, 95% CI 0.97–1.54; *P* = 0.09); Subgroup (stage II) 3-year OS: 75.1% vs. 66.9% (*P* = 0.04).	LN yield (median, IQR): 26.0 (18.0–34.0) vs. 20.0 (14.0–26.0); R0 resection: NR; Pneumonia: 30.1% vs. 26.3%; Anastomotic leak: 9.0% vs. 11.0%; RLN palsy: 26.6% vs. 21.8%; ICU stay: NR; Hospital stay (mean ± SD days): 15.3 ± 9.3 vs. 19.0 ± 10.2; QoL: NR; Major complications (CD ≥ II): 40.0% vs. 36.4%.	Bias: Non-randomized (selection, learning-curve biases); Confounders: 1:1 PSM (gender, age, BMI, ASA-PS, tumor location, cTNM, pTNM); Follow-up: q3mo (2y), then q6mo (to 5y); Analysis: χ², *t*-test; Kaplan–Meier.	30-day mortality: 0.6% vs. 0.6%; 90-day: 0.9% vs. 1.8%; Readmissions: NR; 3-year recurrence: 32.5% vs. 35.5%.
Yamashita et al. 2018 (16), Surg Endosc (Japan)	Type: Retrospective, single-center, propensity-score matched cohort; Funding: NR; Conflict of Interest: none.	N (MIE/OE): 121/121 (PSM); Age (median): 65 vs. 68; Sex (male%): 80.2 vs. 81.8; Stage I/II/III/IV%: 61.2/19.8/17.4/1.7 vs. 53.7/32.2/13.2/0.8; Location (U/M/L%): 14.0/52.9/33.1 vs. 15.7/60.3/24.0; Neoadj (chemo): 45.5% vs. 45.5%; LN field: 2-field or 3-field (supraclavicular).	Approach: MIE: Thoracoscopy (prone) + laparoscopy (or hybrid open); OE: Open right thoracotomy + laparotomy; Conversion: NR; Op time (median, range): 615 (396–956) vs. 490 (310–885) min; Blood loss (median, range): 200 (40–880) vs. 325 (80–2,280) mL.	3-year OS: 89.9% vs. 79.2% (HR OE vs. MIE 2.14, 95% CI 1.19–3.84; *P* = 0.011); 3-year DFS: 81.7% vs. 69.3% (HR OE vs. MIE 1.75, 95% CI 1.07–2.87; *P* = 0.025).	LN yield (total/mediastinal median): 52/21 vs. 56/23; R0 resection: NR; Pulmonary: NR; Anastomotic leak: NR; RLN palsy: NR; ICU stay: NR; Hospital stay (median days): 21 vs. 23; QoL: NR; Major complications (CD ≥ III): 21.5% vs. 18.2%; Peak CRP (median mg/dL): 15.21 vs. 19.50.	Bias: Retrospective, single-center (temporal bias; OE earlier); Confounders: 1:1 PSM (gender, age, CCI, cT/cN/cM, neoadj); Follow-up: median 1,345 vs. 1,912 days; Analysis: χ², Mann–Whitney U; Kaplan–Meier.	30-day mortality: 0% vs. 0%; 90-day: NR; Readmissions: NR; Locoregional recurrence: 5.0% vs. 14.0%; Any recurrence: 15.7% vs. 25.6%.
Wang et al. 2023 (17), Ann Surg (Taiwan)	Type: Retrospective national-registry cohort; Funding: none; Conflict of Interest: none.	N (MIE/OE): 866/433 (matched); Age: 56.5 vs. 57.2; Sex (male%): 92.7 vs. 92.4; Stage 0/I/II/III%: 2.77/33.60/40.76/22.86 vs. 2.54/31.64/38.80/27.02; Location (L/M/U/X%): 26.91/41.69/11.55/19.86 vs. 27.94/42.03/11.78/18.24; Neoadj: none; LN field: NR.	Approach: MIE: Thoracoscopic (Ivor-Lewis or McKeown); OE: Open; Conversion: NR; Op time: NR; Blood loss: NR.	3-year OS (MIE vs. OE): 58.6% vs. 47.6% (*P* = 0.0002); Stage I OS: 76.3% vs. 62.3% (*P* = 0.0032); Stage II OS: 58.8% vs. 46.0% (*P* = 0.0111); Stage III OS: 28.9% vs. 28.7% (*P* = 0.5746); HR (open vs. MIE): 1.24 (95% CI 1.07–1.44; *P* = 0.0041). DFS: NR.	LN yield: NR; R0 resection: 89.4% vs. 88.7%; Pulmonary: NR; Anastomotic leak: NR; RLN injury: NR; ICU stay: NR; Hospital stay: NR; QoL: NR.	Bias: Retrospective registry (heterogeneous protocols); Confounders: 1:2 PSM (age, sex, Charlson, tumor location/length, path T/N/stage, margin, adjuvant CRT); Follow-up: OS to Dec 2017; Analysis: Kaplan–Meier (log-rank); Cox.	30-day: NR; 90-day: NR; Readmissions: NR.
Wang et al. 2022 (18), Ann Thorac Surg (China)	Type: Retrospective cohort; Funding: NR; Conflict of Interest: NR.	N (MIE/OE): 288/288 (matched; pre: 611/306); Age (mean ± SD): 58.4 ± 8.0 vs. 60.1 ± 7.9; Sex (male%): 98.2 vs. 73.6; Stage I/II/III/IV%: 5.6/37.8/55.9/0.7 vs. 5.6/34.4/58.0/2.1; Location (Upper/Mid/Lower%): 13.2/70.8/16.0 vs. 20.5/63.9/15.6; Neoadj: 13.2% vs. 13.5%; LN field: 2-field (incl. bilateral RLNs).	Approach: MIE (McKeown's tMIE): thoracoscopy + laparoscopy; OE (McKeown): open thoracotomy + laparotomy; Conversion: NR; Op time (mean ± SD): 346.9 vs. 362.0 min; Blood loss (mean ± SD): 192.4 vs. 195.0 mL.	OS: Median 61.4 vs. 61.1 months (*P* = 0.545); 5-year OS: 51% vs. 50%; DFS: NR; HRs: NR.	LN yield (mean ± SD): 25.1 vs. 25.6; R0 resection: 96.2% vs. 94.8%; Pneumonia: 7.6% vs. 14.9%; Respiratory insufficiency: 4.9% vs. 11.8%; Cervical leak: 14.2% vs. 27.8%; RLN injury: 6.9% vs. 7.6%; ICU stay (days): 2.4 vs. 3.6; Hospital stay (days): 18.2 vs. 23.2; QoL: NR.	Bias: Single-center retrospective; Confounders: 1:1 PSM (age, sex, BMI, ASA-PS, CCI, tumor location, neoadj, clinical/path stage, tumor differentiation, weight loss); Follow-up: q3mo (yr1), q6mo thereafter; Analysis: Kaplan–Meier, *t*-test/ANOVA, χ²/Fisher.	30-day mortality: 0.0% vs. 2.8% (*P* = 0.004); 90-day: 0.7% vs. 5.9% (*P* < 0.001); Readmissions: NR.
Wang et al. 2015 (19) (J Thorac Cardiovasc Surg, China)	Retrospective cohort; no funding/COI disclosed.	N: pre-match MIE 735 vs. OE 652; post-match MIE 444 vs. OE 444. Age [median (IQR)]: 56 [32–77] vs. 56 [38–76]. Sex (% male): 81.5% vs. 80.6%. Stage (0-I/II/III/IV %): 14.0/57.2/22.5/6.3 vs. 15.5/57.4/22.3/4.7. Location (Upper/Mid/Lower %): 14.2/55.0/30.9 vs. 15.3/56.5/28.2. Neoadj (CRT/Chemo/None): 13.1/5.0/82.0 vs. 11.9/5.0/83.1. LN field: two-field LND (later bilateral).	Approach: MIE (thoracoscopic + laparoscopic/laparotomic) vs. OE (open Ivor-Lewis/McKeown). Conversion: 1.1% (MIE). Operative time: 191 ± 47 vs. 211 ± 44 min (*p* < .001). Blood loss: 135 ± 74 vs. 163 ± 84 mL (*p* < .001).	OS (5-y by stage, post-match): 0-I 78% vs. 78%; II 50% vs. 48%; III 33% vs. 34%; IV 26% vs. 25% (all *p* > 0.5). DFS: NR; HRs: NR.	LN yield: 24.1 ± 6.2 vs. 24.3 ± 6.0 (*p* = 0.607). R0: NR. Pulmonary complications: 8.6% vs. 13.3% (*p* = 0.024). Leak: cervical 11.7% vs. 6.5%; thoracic 0.9% vs. 3.4%. RLN palsy: 5.9% vs. 6.3% (*p* = NR). ICU stay: median 1d vs. 1d (*p* = 0.407). Hospital stay: median 11 vs. 12 d (*p* < 0.001). QoL: significantly better with MIE (*p* < 0.001).	Retrospective single-center; 1:1 PS matching (age, gender, BMI, CCI, ASA, tumor location, cTNM, neoadj, pTNM, period); Follow-up: median 27 mo; Analysis: Kaplan–Meier (log-rank), χ², *t*-test, Fisher's exact, GEE (SPSS).	30d: NR; 90d: NR; Perioperative mortality: 1.1% vs. 2.0% (*p* = 0.281); Readmission: 5.6% vs. 9.7% (*p* = 0.023).
Hamai et al. 2021 (20) (Anticancer Res, Japan)	Retrospective comparative; no funding/COI disclosed.	N: 68 vs. 65. Age (mean ± SD): 65.2 ± 9.0 vs. 64.0 ± 8.8. Sex (% male): 73.5% vs. 86.2%. Stage (cIII/IV %): 69.1 vs. 66.2. Location (Upper/Mid third %): 76.2 vs. 66.1. Neoadj (CRT): 50.0% vs. 56.9%. LN field (3-field): 63.2% vs. 70.8%.	Approach: MIE (prone thoracoscopic + CO₂) vs. OE (open via 4th intercostal thoracotomy). Conversion: none. Operative time (median): thoracic 269 vs. 201 min; total 527 vs. 466 min. Blood loss: 261 vs. 450 g.	OS 5-y: 51.9% vs. 48.9% (*p* = 0.46); DSS 5-y: 59.4% vs. 58.8% (*p* = 0.59); OS HR: 0.83 (95% CI 0.50–1.37, *p* = 0.46).	LN yield: 44.8 ± 16.8 vs. 43.8 ± 16.7 (*p* = 0.71); mediastinal yield: 20.0 ± 8.5 vs. 18.6 ± 9.7 (*p* = 0.31). R0/R1: 98.5% vs. 92.3% (*p* = 0.08). Pneumonia: 7.4% vs. 21.5% (*p* = 0.02). Leak: 19.1% vs. 18.5% (*p* = 0.90). RLN palsy: 22.0% vs. 9.2% (*p* = 0.04). ICU/Hosp stay: NR. QoL: NR.	Retrospective; multivariate analyses for survival and complications; noted differences in abdominal/reconstruction; Follow-up: not reported (survival to 60 mo); Analysis: χ², *t*-test, Kaplan–Meier, logistic regression, Cox (SPSS).	30d: 0% vs. 0%; In-hospital: 0% vs. 1.5% (*p* = 0.30); 90d: NR; Readmission: NR.

MIE, minimally invasive esophagectomy; OE, open esophagectomy; RAMIE, robot-assisted minimally invasive esophagectomy; tMIE, totally minimally invasive esophagectomy; TE, thoracoscopic esophagectomy; HALS, hand-assisted laparoscopic surgery; HR, hazard ratio; CI, confidence interval; OS, overall survival; DFS, disease-free survival; PFS, progression-free survival; CSS, cancer-specific survival; DSS, disease-specific survival; LN, lymph node; LND, lymph node dissection; RLN, recurrent laryngeal nerve; R0, resection with microscopically negative margins; R1, resection with microscopically positive margins; ICU, intensive care unit; QoL, quality of life; NR, not reported; N/A, not applicable; IQR, interquartile range; Neoadj, neoadjuvant therapy; Chemo, chemotherapy; CRT, chemoradiotherapy; ASA-PS, American Society of Anesthesiologists physical status; BMI, body mass index; CCI, Charlson comorbidity index; PSM, propensity score matching; IPTW, inverse probability of treatment weighting; SMD, standardized mean difference; CD, Clavien–Dindo classification; CT, computed tomography; CRP, C-reactive protein; IL, interleukin; cTNM, clinical tumor–node–metastasis stage; pTNM, pathologic tumor–node–metastasis stage; U/M/L, upper/middle/lower esophagus; 30d/90d, 30-day/90-day mortality.

### Data Synthesis Approach

Given the methodological heterogeneity in surgical techniques and outcome reporting, a narrative synthesis was performed rather than meta-analysis. Survival outcomes were stratified by tumor location (upper/middle esophagus), where data permitted. Complication rates were analyzed according to Clavien-Dindo classification when available, with subgroup consideration for anastomotic technique (cervical vs. intrathoracic). Sensitivity analyses excluded studies with significant neoadjuvant therapy imbalances or high risk of bias. The Grading of Recommendations Assessment, Development and Evaluation (GRADE) framework was applied to evaluate the overall strength of evidence for each outcome domain.

## Result

### Oncologic and survival outcomes

Across all nine studies, oncologic metrics were generally comparable between MIE and OE. R0 resection rates were uniformly high (typically ≥92% in both groups). Lymph node harvests were similar as well; for example, Yun et al. found mean yields of 39.1 vs. 38.3 (MIE vs. OE), and Mao et al. reported medians of 26 vs. 20. Survival outcomes showed modest differences (12, 15). Of the nine included studies, six (66.7%) reported no statistically significant difference in overall survival (OS) between MIE and OE (12, 13, 16, 18–20). For example, Yun et al. observed nearly overlapping 3-year OS (81.7% vs. 73.7%, *p* = 0.05) (12), and Hamai et al. reported 5-year OS of 51.9% vs. 48.9% (*p* = 0.46) (20). Two studies (22.2%) reported significantly better OS with MIE (14, 17), including Terayama et al. (HR 0.54, 95% CI 0.43–0.68, *p* < 0.001) and Wang et al. (2023) (3-year OS 58.6% vs. 47.6%, *p* = 0.0002). One study (11.1%) reported a paradoxical, slightly inferior 3-year OS with MIE (HR = 1.33, *p* = 0.03) (15). A similar distribution was seen for disease-free survival (DFS), with four out of seven (44.4%) reporting DFS, finding no significant difference (12, 13, 15, 20). One study (11.1%) reported significantly better DFS with MIE (HR 1.75, *p* = 0.025) (16) (Table 3).

**Table 3:** Oncologic and survival outcomes (MIE vs. OE).

Outcome	Yun et al. 2020 (12) (Korea)	Kanekiyo et al. 2018 (13) (Japan)	Terayama et al. 2024 (14) (Japan)	Mao et al. 2023 (15) (China)	Yamashita et al. 2018 (16) (Japan)	Wang et al. 2023 (17) (Taiwan)	Wang et al. 2022 (18) (China)	Wang et al. 2015 (19) (China)	Hamai et al. 2021 (20) (Japan)
R0 resection (MIE vs. OE)	127/130 (97.7%) vs. 233/241 (96.7%); *p* = 0.719	NR	641/651 (98.6%) vs. 354/382 (92.7%); *p* < 0.001	NR	NR	774/866 (89.38%) vs. 384/433 (88.68%)	277/288 (96.2%) vs. 273/288 (94.8%); 0.422	NR	67/68 (98.5%) vs. 60/65 (92.3%)
Lymph node yield (mean/median) (MIE vs. OE)	39.1 ± 13.8 vs. 38.3 ± 12.9; *p* = 0.571	Mediastinal median 25 (20–30) vs. 21 (16–28); *p* = 0.030	NR	Total median 26 (18–34) vs. 20 (14–26); *p* < 0.001	Total median 52 (23–118) vs. 56 (12–109); *p* = 0.14. mediastinal 21 (6–56) vs. 23 (1–52); *p* = 0.38	NR	25.1 ± 10.5 vs. 25.6 ± 12.0; *p* = 0.639	24.1 ± 6.2 vs. 24.3 ± 6.0; *p* = 0.607	44.8 ± 16.8 vs. 43.8 ± 16.7; *p* = 0.71
Overall survival (OS) (MIE vs. OE)	1-yr OS 124/130 (95.1%) vs. 206/241 (85.6%); 3-yr OS 106/130 (81.7%) vs. 178/241 (73.7%); *p* = 0.051	5-yr OS 42/65 (64.9%) vs. 33/65 (50.2%); *p* = 0.101	3-yr OS HR (MIE vs. OE) 0.54 (95% CI 0.43–0.68); *p* < 0.001	3-yr OS 258/335 (77.0%) vs. 231/335 (69.0%); HR (MIE vs. OE) 1.33 (95% CI 1.02–1.73), *p* = 0.03	3-yr OS 109/121 (89.9%) vs. 96/121 (79.2%); multivariable HR (OE vs. MIE): 2.14 (1.19–3.84), *p* = 0.011	3-yr OS 507/866 (58.58%) vs. 206/433 (47.62%); *p* = 0.0002	5-yr OS 51% vs. 50% (*p* = 0.545)	By stage, 2- and 5-year OS showed no significant differences: stage 0/I (92% vs. 90%, *P* = .864), II (83% vs. 82%, *P* = .725), III (59% vs. 55%, *P* = .592), and IV (43% vs. 43%, *P* = .802).	
	5-yr OS 35/68 (51.9%) vs. 32/65 (48.9%); *p* = 0.46								
Disease-free survival (DFS)/progression-free survival (PFS)/disease-specific survival (DSS) (MIE vs. OE)	1-yr DFS 71/130 (54.4%) vs. 128/241 (53.2%); 3-yr DFS 64/130 (49.2%) vs. 110/241 (45.6%); *p* = 0.217	5-yr PFS 46/65 (70.6%) vs. 38/65 (58.7%); *p* = 0.328	NR	3-yr DFS 228/335 (68.1%) vs. 204/335 (60.9%); HR 1.22 (0.97–1.54), *p* = 0.09	3-yr DFS 99/121 (81.7%) vs. 84/121 (69.3%); multivariable HR (OE vs. MIE) 1.75 (1.07–2.87), *p* = 0.025	NR	NR	NR	5-yr DSS 40/68 (59.4%) vs. 38/65 (58.8%); *p* = 0.59

MIE, minimally invasive esophagectomy; OE, open esophagectomy; HR, hazard ratio; CI, confidence interval; OS, overall survival; PFS, progression-free survival; DFS, disease-free survival; DSS, disease-specific survival; IQR, interquartile range; NR, not reported; R0, resection with microscopically negative margins.

### Recovery and perioperative outcomes

Minimally invasive approaches consistently shortened recovery. Operative time was longer for MIE in six of nine studies (66.7%). For example, Yun et al. reported a mean operative time of 275.6 ± 71.1 min for MIE vs. 240.0 ± 48.9 min for OE (*p* < 0.001), while Kanekiyo et al. reported median operative times of 536 min for MIE vs. 491 min for OE (*p* < 0.001) (12, 13). Reduced intraoperative blood loss with MIE was reported in eight of nine studies (88.9%), with one study (11.1%) reporting no significant difference (12–16, 18–20). Shorter ICU and/or hospital stays were observed in all seven studies reporting these outcomes, including Wang et al. (2022), who found significantly shorter ICU (2.4 ± 4.1 vs. 3.6 ± 5.9 days, MIE vs. OE; *p* = 0.007) and hospital stays (18.2 ± 16.5 vs. 23.2 ± 21.7 days, MIE vs. OE; *p* = 0.002) with MIE (18) (Table 4).

**Table 4:** Recovery and perioperative outcomes (MIE vs. OE).

Outcome/study	Yun et al. 2020 (12)	Kanekiyo et al. 2018 (13)	Terayama et al. 2024 (14)	Mao et al. 2023 (15)	Yamashita et al. 2018 (16)	Wang et al. 2023 (17)	Wang et al. 2022 (18)	Wang et al. 2015 (19)	Hamai et al. 2021 (20)
Operative time (mean/median) (MIE vs. OE)	275.6 ± 71.1 min vs. 240.0 ± 48.9 min; *p* = 0.001	536 (501–593) min vs. 491 (415–575) min; *p* < 0.001	540 (259–957) vs. 492 (260–1,047) min; *p* < 0.001	279 ± 93.5 vs. 277 ± 81.2 min; *p* = 0.662	615 (396–956) vs. 490 (310–885) min; *p* < 0.001	NR	346.9 ± 111.6 vs. 362.0 ± 130.3 min; *p* = 0.620	191 ± 47 vs. 211 ± 44 min; *p* < 0.001	527 (350–747) min vs. 466 (329–1,003) min; *p* = 0.02
Blood loss (mean/median) (MIE vs. OE)	110.8 ± 125.8 mL vs. 93.8 ± 140.9 mL; *p* = 0.251	250 (160–503) mL vs. 599 (360–875) mL; *p* < 0.001	110 (15–880) mL vs. 380 (50–3,250) mL; *p* < 0.001	162 ± 196 mL vs. 227 ± 144 mL; *p* < 0.001	200 (40–880) mL vs. 325 (80–2,280) mL; *p* < 0.001	NR	192.4 ± 172.0 mL vs. 195.0 ± 122.9 mL; *p* = 0.830	135 ± 74 mL vs. 163 ± 84 mL; *p* < 0.001	261 (57–1,912) g vs. 450 (195–2,030) g; *p* = 0.0,004
ICU stay (mean) (MIE vs. OE)	1.08 ± 0.43 days vs. 1.36 ± 1.97 days; *p* = 0.122	NR	NR	NR	NR	NR	2.4 ± 4.1 days vs. 3.6 ± 5.9 days, *p* = 0.007	1 day (0–30) vs. 1 day (0–39); *p* = 0.407	NR
Hospital stays (postoperative) (mean/median) (MIE vs. OE)	16.5 ± 9.8 days vs. 18.2 ± 15.4 days; *p* = 0.265	29 (22–41) days vs. 35 (25–66) days; *p* = 0.038	20 (12–149) vs. 22 (12–457) days; *p* < 0.001	15.3 ± 9.3 days vs. 19.0 ± 10.2 days; *p* < 0.001	21 (13–457) vs. 23 (15–138) days; *p* = 0.38	NR	18.2 ± 16.5 days vs. 23.2 ± 21.7 days, *p* = 0.002	11 (7–90) vs. 12 (8–112) days, *p* < 0.001	NR
30-day mortality (MIE vs. OE)	30-day: 0/130 (0%) vs. 4/241 (1.7%); *p* = 0.342	30-day: 0/65 (0%) vs. 0/65 (0%)	30-day: 1/651 (0.1%) vs. 1/382 (0.3%); *p* = 1.000	30-day: 2/335 (0.6%) vs. 2/335 (0.6%); *p* = 0.998	NR	NR	30-day: 0/288 (0%) vs. 8/288 (2.8%), *p* = 0.004	30-day: 5/444 (1.1%) vs. 9/444 (2.0%), *p* = 0.281	30-day: 0/68 (0%) vs. 0/65 (0%)

MIE, minimally invasive esophagectomy; OE, open esophagectomy; min, minutes; mL, milliliters; ICU, intensive care unit; d, days; vs., versus; NR, not reported; IQR, interquartile range.

### Pulmonary and other complications

Pulmonary complications, particularly pneumonia, occurred less frequently after MIE in seven of nine studies (77.8%). For instance, pneumonia rates were 3.8% vs. 10.8% (MIE vs. OE) in Yun et al., 16.9% vs. 33.9% (MIE vs. OE) in Kanekiyo et al., and 7.6% vs. 14.9% (MIE vs. OE; *p* = 0.006) in Wang et al. (12, 13, 18). Two studies (22.2%) reported similar or slightly higher pulmonary complication rates with MIE (14, 15). Anastomotic leak rates were similar between approaches in seven of nine studies (77.8%), with one study (11.1%) reporting a lower cervical leak rate with MIE (14.2% vs. 27.8%, MIE vs. OE; *p* < 0.001) (18). Recurrent laryngeal nerve injury showed variable results, with two studies (22.2%) reporting higher rates following MIE and the remainder showing no significant differences (12, 20) (Table 5).

**Table 5:** Pulmonary and other complications (MIE vs. OE).

Outcome/study	Yun et al. 2020 (12)	Kanekiyo et al. 2018 (13)	Terayama et al. 2024 (14)	Mao et al. 2023 (15)	Yamashita et al. 2018 (16)	Wang et al. 2023 (17)	Wang et al. 2022 (18)	Wang et al. 2015 (19)	Hamai et al. 2021 (20)
Pneumonia/pulmonary complications (MIE vs. OE)	5/130 (3.8%) vs. 26/241 (10.8%); *p* = 0.035	11/65 (16.9%) vs. 22/65 (33.9%); *p* = 0.043	118/651 (18.1%) vs. 70/382 (18.3%) (CD ≥2); *p* = 0.931	101/335 (30.1%) vs. 88/335 (26.3%); *p* = 0.303	NR	NR	22/288 (7.6%) vs. 43/288 (14.9%), *p* = 0.006	38/444 (8.6%) vs. 59/444 (13.3%), *p* = 0.024	5/68 (7.4%) vs. 14/65 (21.5%), *p* = 0.02
Anastomotic leak (overall/by site) (MIE vs. OE)	4/130 (3.1%) vs. 7/241 (2.9%); *p* = 0.942	7/65 (10.8%) vs. 8/65 (12.3%); *p* = 1.000	70/651 (10.7%) vs. 31/382 (8.3%); *p* = 0.193	30/335 (9.0%) vs. 37/335 (11.0%); *p* = 0.44	NR	NR	Cervical leak: 41/288 (14.2%) vs. 80/288 (27.8%), *p* < 0.001	Cervical leak 52/444 (11.7%) vs. 29/444 (6.5%); Intrathoracic 4/444 (0.9%) vs. 15/444 (3.4%); *p* = 0.308	13/68 (19.1%) vs. 12/65 (18.5%), *p* = 0.90
Recurrent laryngeal nerve (RLN) palsy/hoarseness (MIE vs. OE)	(25%) vs. (19%); *p* = 0.278	15/65 (23.1%) vs. 19/65 (29.3%); *p* = 0.550	26/651 (4.0%) vs. 12/382 (3.1%); *p* = 0.608	89/335 (26.6%) vs. 73/335 (21.8%); *p* = 0.176	NR	NR	20/288 (6.9%) vs. 22/288 (7.6%), *p* = 0.749	26/444 (5.9%) vs. 28/444 (6.3%)	15/68 (22.0%) vs. 6/65 (9.2%), *p* = 0.04

MIE, minimally invasive esophagectomy; OE, open esophagectomy; RLN, recurrent laryngeal nerve; vs., versus; NR, not reported.

## Discussion

This systematic review of nine contemporary comparative studies provides a focused analysis of the outcomes of minimally invasive (MIE) vs. open esophagectomy (OE) in patients with esophageal squamous cell carcinoma (ESCC). Our findings demonstrate that MIE achieves oncologic outcomes comparable to OE while conferring significant advantages in postoperative recovery and reducing specific complications.

In line with large meta-analyses and randomized trials, our analysis confirms that MIE does not compromise key oncologic metrics (22, 23). R0 resection rates were uniformly high and equivalent between the two groups, and lymph node harvests were similar, underscoring the oncologic adequacy of the minimally invasive approach. Regarding long-term survival, the pooled results were largely comparable, with most studies reporting no significant difference in overall survival (OS) or disease-free survival (DFS). For instance, Yun et al. and Hamai et al. found no statistically significant difference in 3-year and 5-year OS, respectively (12, 20). Notably, some studies even suggested a potential survival benefit for MIE, with Terayama et al. and Wang et al. reporting significantly better 3-year OS (14, 17). Only one study (Mao et al.) paradoxically found slightly inferior 3-year OS with MIE (15). This overall pattern of equivalence or potential benefit aligns with prior literature; the TIME randomized trial, for example, showed virtually identical 3-year survival between approaches (23), and Wang et al. similarly reported no significant OS difference at 5 years (24). Thus, our review robustly supports the consensus that MIE can achieve oncological outcomes equivalent to, and potentially superior to, OE in ESCC.

A consistent and notable trend in our results was the reduction in pulmonary morbidity associated with MIE. Most included studies reported lower rates of pneumonia or pulmonary complications in the MIE cohort. For example, Yun et al., Kanekiyo et al., and Hamai et al. documented significant reductions, while others showed non-significant favorable trends (12, 13, 20). This finding is a cornerstone of the MIE benefit profile and aligns perfectly with multiple meta-analyses, including a pooled analysis of approximately 15,000 patients that found a 47% reduction in pulmonary complications for MIE (22). The TIME trial likewise documented significantly fewer postoperative respiratory infections with the minimally invasive approach (23). In contrast, rates of other complications, such as anastomotic leak, were largely similar between groups, consistent with prior data (22). Overall, major complication rates in our review were largely equivalent, reinforcing the evidence that MIE does not increase perioperative risk and may in fact reduce specific, serious morbidities (24).

Regarding postoperative recovery, our analysis reveals meaningful benefits for patients undergoing MIE. Operative time was generally longer for MIE, but this was counterbalanced by significantly reduced blood loss. More importantly, MIE was consistently associated with shorter intensive care unit and overall hospital stays across all studies reporting these metrics. For instance, Wang et al. found significantly shorter ICU and hospital stays with MIE, and Wang et al. reported a median postoperative stay of 11 (7–90) days for MIE vs. 12 (8–112) days for OE (*p* < 0.001) (18, 19). These findings echo those from randomized trials and meta-analyses; Yibulayin et al. similarly concluded that MIE yields shorter hospital stays and less blood loss, and the landmark RCT by Biere et al. demonstrated reduced pulmonary complications and improved short-term quality of life with MIE (22, 25). The perioperative advantages of reduced surgical trauma, less blood loss, and shorter convalescence, coupled with equivalent oncologic efficacy, suggest that MIE offers a superior short-term outcome profile without compromising cancer control.

These findings have important clinical and research implications. Clinically, they support the safety and efficacy of MIE for ESCC, indicating that high-volume centers with expertise in minimally invasive techniques can adopt MIE as a standard of care. The consistency of our results with prior high-quality literature, including randomized trials like TIME and MIRO, lends considerable confidence to this recommendation (22, 23). For research, our review highlights the need for further prospective, ideally randomized studies focused exclusively on ESCC, as most existing RCTs have included mixed histologies or focused on adenocarcinoma. Future work should also strive to better quantify patient-centered outcomes, such as quality of life and long-term functional morbidity, following MIE for ESCC.

This review has limitations, primarily stemming from the retrospective and observational design of all included studies. Despite the use of propensity-score methods, residual selection bias and unmeasured confounding remain possible. Furthermore, heterogeneity in surgical techniques (e.g., robotic-assisted vs. conventional MIE, variations in anastomotic approach) and perioperative care pathways could influence the outcomes. Nevertheless, the congruence of our systematic analysis with external evidence from meta-analyses and RCTs suggests that our conclusions are robust.

## Conclusion

Minimally invasive esophagectomy (MIE) demonstrates oncological outcomes comparable to those of open esophagectomy for patients with esophageal squamous cell carcinoma (ESCC), reflected in similar rates of R0 resection, lymph node yield, and overall and disease-free survival. Beyond equivalent oncologic efficacy, MIE is consistently associated with lower pulmonary morbidity, reduced intraoperative blood loss, and shorter postoperative length of stay—clinically meaningful differences. These concordant findings support the formal integration of MIE into contemporary multidisciplinary treatment algorithms and its consideration in guideline revisions, while emphasizing that optimal results require rigorous patient selection, procedural standardization, and adequate surgeon and institutional expertise.

## Data Availability

The original contributions presented in the study are included in the article/Supplementary Material, further inquiries can be directed to the corresponding author.
